# Supplementary material for: Expert consensus on pre-eclampsia risk screening tools for low- and middle-income countries: Development of a new Target Product Profile
Source: PLOS Glob Public Health. 2026 Mar 2;6(3):e0005766. doi: 10.1371/journal.pgph.0005766 (PMC12952618; doi:10.1371/journal.pgph.0005766)
Supplement: S1 Appendix — (DOCX) [file pgph.0005766.s001.docx]

## S1 Appendix: Strategies to identify stakeholders from each group

| ***Stakeholder groups*** | ***Methods of stakeholder identification*** | ***Method of survey distribution*** | ***Additional methods of distribution*** |
| --- | --- | --- | --- |
| Obstetricians with special interest/expertise in pre-eclampsia | - Via listservs of special societies/professional groups (FIGO, FLASOG, AOFOG and AFOG). | - Direct email contact | - Distribution via the listservs of Jhpiego - Distribution of survey via social media and newsletter of Perinatal Society of Australia and New Zealand (PSANZ) |
| Midwives and nurses | - Via listservs of international nursing and midwifery societies by engaging with main contact (ICM, INF, PSANZ and others). | - Direct email contact | - Distribution of survey via social media and newsletter of PSANZ, the John Hopkins Program for International Education in Gynecology and Obstetrics and the Australian Action on Pre-eclampsia. - Distribution via the listservs of Jhpiego |
| Academics / researchers working on pre-eclampsia | - Systematic identification of top 50 researchers for key terms* through SCOPUS, with direct email contact | - Direct email contact | - Distribution of survey via social media and newsletter of PSANZ |
| Global diagnostics and innovation representatives | - Contacting leading representatives from relevant special societies/international groups (e.g. PATH, FIND) | - Distribution of survey via master list of global diagnostics and innovation representatives developed through literature review | - Distribution of survey via listservs of FIND |
| Antenatal care program managers | - Through contacting representatives from various WHO Country Offices, for further distribution to National Ministry of Health’s ANC program manager networks | - Direct email contact |  |
| Maternal diagnostics manufacturers | - Online searching to develop list of consumer organizations interested in pre-eclampsia and maternal health - Analysis of 2024 Maternal Health Pipeline | - Distribution of survey to a master list of maternal diagnostics manufacturers developed through literature review |  |
| Consumer representatives | - Online searching to develop list of consumer organizations interested in pre-eclampsia and maternal health | - Direct email contact |  |
| Guideline panel members | - Literature review to identify and contact all guideline panel members from WHO, NICE, ACOG and other relevant guidelines relating to pre-eclampsia and pre-eclampsia risk screening from the last 10 years | - Direct email contact |  |
| Procurement experts | - Via listservs of specific organisations (e.g. Reproductive Health Supplies Coalition) | - Direct email contact |  |
| International health agency or organization staff | - WHO and UNFPA staff working on maternal health at Headquarters, Regional and Country Office levels - Web searches to identify other international non-governmental organizations | - Direct email contact | - Distribution via the listservs of Jhpiego |

*The top 50 researchers on Scopus were identified across five categories with the following search terms: ‘Pre-eclampsia risk screening”, “Pre-eclampsia diagnostics”, “Pre-eclampsia”, “Maternal Diagnostics” and “antenatal diagnostic tests”.
