# Supplementary material for: Expert consensus on pre-eclampsia risk screening tools for low- and middle-income countries: Development of a new Target Product Profile
Source: PLOS Glob Public Health. 2026 Mar 2;6(3):e0005766. doi: 10.1371/journal.pgph.0005766 (PMC12952618; doi:10.1371/journal.pgph.0005766)
Supplement: S2 Appendix — (DOCX) [file pgph.0005766.s002.docx]

## S2 Appendix: Interview guide:

| Interviewer/s |  |
| --- | --- |
| Date |  |
| Start and finish time |  |
| Participant code |  |
| Participant field |  |

Preamble:

Welcome. Thank you for joining the interview.

The purpose of the interview is to discuss your opinions on the new target product profile (TPP) that we have developed for pre-eclampsia risk screening tools.

This TPP is part of a much wider project called the AIM project – accelerating innovation for mothers – where multiple TPPs have already been published. Currently no TPP exists on risk screening tools for pre-eclampsia.

To recap, a target product profile is a written protocol which lists the minimum and optimal characteristics a product should take, to fulfil a specific clinical or public health need. Minimum targets are those which the product must address in order to meet the specific need whereas optimistic targets are ideal/desirable. So to clarify, because this is often an area of confusion amongst many, our end product will be a written document which guides development of the pre-eclampsia risk screening tool but not the risk screening tool itself.

You will have seen in the draft version shared with you, that the TPP includes a brief description of the problem, the scenario in which interventions would be used, and the large table of variables with the minimum and optimal characteristics. We will go through these sections today and ask you whether you agree or disagree with what has been written. We are expecting the draft to change with the feedback and so we warmly welcome all feedback.

Lastly, I just want to go over what we mean by tool. We feel that this terminology best captures the range of tests or methods that can either be used alone or together to predict risk of pre-eclampsia.

Do you have any questions before we proceed?

Housekeeping:

- I will be taking notes to keep record of your feedback.
- We would also like to ask your permission to record this interview, so that we may refer to it if needed. The recording will not be shared outside the research team and will be destroyed at the completion of the study.
- All the comments you provide will be treated as non-identifiable, so they will not be attributed to you by name or released outside of our study team.
- Any questions or variables that you do not have any feedback on, do not have to be answered.

Verbal consent to participate

Verbal consent to record interview ~start recording interview~

I will now ask questions on the TPP, trying to gauge whether you agree or disagree, and any comments or suggestions you may have.

| Q1: Reading the background/preamble, is the problem definition in this TPP clear? It is clear? Was there anything missing? Ask about whether treatment of pre-eclampsia should be mentioned (no cure but treatment is delivery of baby).  Starts page 2 |
| --- |
|  |
| Q2: Do you agree with the wording of the intended use case scenario? Should explain when and by who it should be used. What changes, if any, need to be made to the use case scenario?  Starts page 4 |
|  |
| Q3: Jumping into the TPP variable table, we would like to discuss ***(insert relevant variable)*** with you, given your expertise. Are there any other variables on which you would like to share your thoughts?  Will now move to the body of the TPP table. Were there any variables you felt particularly strongly on that you would like to start on, or alternatively I can work through these one-by-one.  Do you agree with X variable  Do you agree with wording of X variable  Was there anything missing from X variable  For X variable, would you agree with both minimum and optimistic  When you look at X variable, do you agree with what we have put for minimum and optimistic, and the difference between the two?  If we look at X variable, do you agree with the content included here.  This was a variable where there was a lot of a back and forth. We have read literature and arrived at xxx based on comparing with risk screening tools. But what are your thoughts? |
| - Intended use - Target Population: - Target countries: - Target users: - Design and Functionality: offline use in minimum target - Acceptability: - Tool validation: tool has been externally validated in at least one target country by an independent group (not the developer) - Regulation: - Procurement Price: no more than 10US cents - Primary Target Delivery Channel: - Packaging: - Environmental stability: - Training requirements: - External Support: - Device-based technologies: (ask about AI technology) - Point of care tests: - Sample types, collection and processing: lab staff and equipment should not be included in minimum, non invasive samples for optimistic. - Clinical Specificity and Sensitivity; - Safety - Calculation of risk - Results - Time to result: - Tool recommendations: function of the ministry of health, but too much of a burden on the health developer. - Data input - Data security and privacy   This covers all variables in the TPP. Briefly some further, broader questions |
| Q4: Are there any domains that you were uncomfortable with? |
|  |
| Q5: Do you see any significant gaps in the TPP? |
|  |
| Q6: Do you think that this TPP is designed to be applicable to all countries and income settings? |
|  |
| Q7: Could you suggest improvements, both for the content and structure of the document? (Was the content clear and easy to digest?) |
|  |
| Q8: What do you think are ways in which you think the TPP can be more widely disseminated How do you think we can make improvements in how the TPP is disseminated other than the traditional forms |

Additional comments:
