# Supplementary material for: Expert consensus on pre-eclampsia risk screening tools for low- and middle-income countries: Development of a new Target Product Profile
Source: PLOS Glob Public Health. 2026 Mar 2;6(3):e0005766. doi: 10.1371/journal.pgph.0005766 (PMC12952618; doi:10.1371/journal.pgph.0005766)
Supplement: S3 Appendix — (DOCX) [file pgph.0005766.s003.docx]

Pre-eclampsia Risk Screening TPP

Start of Block: Block 1

Q1 Concept Foundation and Burnet Institute have partnered to develop a new Target Product Profile (TPP) for pre-eclampsia risk screening. A TPP describes the key characteristics (minimum and preferred) that an intervention - such as a pre-eclampsia risk screening tool - must address to meet health needs. TPPs are an important resource for multiple stakeholders in the research and development (R&D) pathway, including funders, researchers, product developers, manufacturers, and regulators. The purpose of this survey is to collect input from diverse stakeholders on the development of a new TPP for a pre-eclampsia risk screening tool. This TPP will be revised based on responses to this survey from key stakeholders internationally. The goal of this survey is to define the characteristics of a tool for pre-eclampsia risk screening amongst pregnant women that best meets global health needs. As an important stakeholder, we need your input on this TPP. The survey will take approximately 15 minutes to complete, and all responses are anonymous. You are welcome to skip any question you do not feel able to answer. If you have any questions regarding this survey, please contact: Tahlia Guneratne (tahlia.guneratne@burnet.edu.au);  Dr Annie McDougall (annie.mcdougall@burnet.edu.au); or Dr Joshua Vogel (joshua.vogel@burnet.edu.au) By clicking to the next page, you are indicating your consent to participate.

| Page Break |  |
| --- | --- |

Q101 **Background** Pre-eclampsia is a complication of pregnancy that is characterised by the presence of hypertension, proteinuria and/or end organ dysfunction, presenting after 20 weeks' gestation. It is a leading cause of global maternal and neonatal mortality with an estimated 4.6% of pregnant women experiencing the condition.  Screening methods that can accurately identify women at increased risk of developing pre-eclampsia are essential. They permit the timely initiation of preventative therapies (such as low-dose aspirin) as well as enhanced antenatal monitoring. Historically, pre-eclampsia risk screening has been based on selected maternal characteristics and past medical history, though this approach does not detect many women at high risk. However, other screening methods have recently emerged and have proven more accurate in predicting risk for pre-eclampsia. Pre-eclampsia risk screening methods based on maternal history remain routine in many settings, including in low-resource countries. This is because there are multiple barriers to implementing certain pre-eclampsia risk screening methods at scale. Reasons include insufficient infrastructure, limitations in the available health and laboratory workforce, and many women not commencing antenatal care in early pregnancy.  Therefore, innovations in pre-eclampsia risk screening are needed – particularly those that are accurate, affordable, sustainable and best meet the needs of pregnant women in diverse settings.

| Page Break |  |
| --- | --- |

Q113 **What is a pre-eclampsia risk screening tool?** There are a wide range of methods for pre-eclampsia risk screening and prediction.   Single parameter methods use one type of test to determine risk for pre-eclampsia. For example, a sample of blood or urine which can be tested for a predictive biomarker.   Other methods may use a combination of multiple types of tests to determine risk for pre-eclampsia, known as a multiparametric method. For example, information on maternal history, a biomarker test result (via blood or urine sample) and a machine-based test (eg. an ultrasound to measure uterine artery pulsatility index). These different results may be combined using an algorithm to produce a woman’s risk level or risk score. By ‘risk screening tool’, we mean any method for pre-eclampsia risk screening and prediction, whether single or multiple parameter.

End of Block: Block 1

Start of Block: Section 1: Demographics

Q2 Please select which of the following positions best describes your current role.

- Academic researcher (1)
- Antenatal care program manager (5)
- Consumer representative (2)
- Global diagnostics and innovation representatives (13)
- Guideline panel member (3)
- International health agency or organization staff (4)
- Maternal diagnostics manufacturers (9)
- Nurse/Midwife (6)
- Obstetrician (7)
- Procurement expert (11)
- Other (please specify): (12) __________________________________________________

| 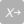 |
| --- |

Q3 In which country do you currently reside?

▼ Afghanistan (1) ... Zimbabwe (1357)

Q4 What is your gender?

- Woman (1)
- Man (2)
- Non-binary / gender diverse (3)
- Prefer to self-describe (4) __________________________________________________
- Prefer not to say (5)

Q5 Overall, please indicate how many years of professional experience you have in your field.

- None (1)
- Less than one year (2)
- 1-5 years (3)
- 5-10 years (4)
- More than 10 years (5)

Q6 Please indicate how familiar you feel you are with the concept of Target Product Profiles?

- Not familiar at all (1)
- Somewhat familiar (2)
- Moderately familiar (3)
- Very familiar (4)
- Extremely familiar (5)

| Page Break |  |
| --- | --- |

Q7 Please indicate if you have a commercial or financial interest in the development of pre-eclampsia risk screening tools. For example, if you hold, or previously have held, a role within a maternal diagnostics manufacturing company (including in a consulting capacity).

- Yes, I have a commercial or financial interest in the development of pre-eclampsia risk screening tools. (1)
- No, I do NOT have a commercial or financial interest in the development of pre-eclampsia risk screening tools. (2)

End of Block: Section 1: Demographics

Start of Block: Section 2: Rating your agreement with the Target Product Profiles

Q1 The following sections will provide a Minimum and Optimistic variable for each of the 25 TPP domains, along with annotations to clarify meaning. The "Minimum target" should be considered as a potential go/no go decision point. The "Optimistic target" should reflect what is needed to achieve broader, deeper global health impact. Please rate your agreement or disagreement for each. An additional section for any comments you may have is at the end of each page.

| Page Break |  |
| --- | --- |

Q2 **Variable 1: What is the intended use of a pre-eclampsia risk screening tool?** *Pre-eclampsia risk screening tools should identify those at high risk of developing pre-eclampsia so that they can benefit from timely initiation of preventative therapies, such as low-dose aspirin, and enhanced antenatal monitoring.*

Q3 **Minimum target:** A tool for screening pregnant women to identify those at high risk of developing pre-eclampsia who can benefit from timely initiation of preventative therapies.

- Strongly agree (5)
- Mostly agree (4)
- Neither agree nor disagree (3)
- Mostly disagree (2)
- Strongly disagree (1)
- No answer (6)

Q4 **Optimistic target:** Same as minimum. *(A tool for screening pregnant women to identify those at high risk of developing pre-eclampsia who can benefit from timely initiation of preventative therapies.)* Plus: Tool can predict and distinguish between onset of pre-eclampsia (such as early onset, late onset, preterm, term).

- Strongly agree (5)
- Mostly agree (4)
- Neither agree nor disagree (3)
- Mostly disagree (2)
- Strongly disagree (1)
- No answer (6)

Q5 Additional comments*(please provide any further comments you may have on this variable)*

________________________________________________________________

________________________________________________________________

________________________________________________________________

________________________________________________________________

________________________________________________________________

| Page Break |  |
| --- | --- |

Q6 **Variable 2: What is the target population for a pre-eclampsia risk screening tool?** *Risk screening for pre-eclampsia should be conducted for all pregnant women as early as possible and ideally before 20 weeks’ gestation. However, some women will not have access to antenatal services early in pregnancy. Therefore, a risk screening tool should still be able to accurately predict risk of pre-eclampsia for women presenting to antenatal care later in pregnancy.*

Q7 **Minimum target:** All pregnant women, adolescent girls, and transgender and gender diverse people. Tool can be used during pregnancy, including in the first trimester.

- Strongly agree (5)
- Mostly agree (4)
- Neither agree nor disagree (3)
- Mostly disagree (2)
- Strongly disagree (1)
- No answer (6)

Q8 **Optimistic Target:** Same as minimum. *(All pregnant women, adolescent girls, and transgender and gender diverse people. Tool can be used during pregnancy, including in the first trimester.)*

- Strongly agree (5)
- Mostly agree (4)
- Neither agree nor disagree (3)
- Mostly disagree (2)
- Strongly disagree (1)
- No answer (6)

Q9 Additional comments *(please provide any further comments you may have on this variable)*

________________________________________________________________

________________________________________________________________

________________________________________________________________

________________________________________________________________

________________________________________________________________

| Page Break |  |
| --- | --- |

Q10 **Variable 3: What are the target countries for a pre-eclampsia risk screening tool?** *Pre-eclampsia can affect women of any background, or in any setting. The highest rates of pre-eclampsia are observed in Africa, Europe and South-East Asia. A pre-eclampsia risk screening tool must be usable across high-, medium- and low-resource settings.*

Q11 **Minimum target:** All countries, with a particular focus on limited-resource settings and regions with high rates of pre-eclampsia and/or poor maternal and neonatal health outcomes related to pre-eclampsia.

- Strongly agree (5)
- Mostly agree (4)
- Neither agree nor disagree (3)
- Mostly disagree (2)
- Strongly disagree (1)
- No answer (6)

Q12 **Optimistic Target:** Same as minimum. *(All countries, with a particular focus on limited-resource settings and regions with high rates of pre-eclampsia and/or poor maternal and neonatal health outcomes related to pre-eclampsia.)*

- Strongly agree (5)
- Mostly agree (4)
- Neither agree nor disagree (3)
- Mostly disagree (2)
- Strongly disagree (1)
- No answer (6)

Q13 Additional comments *(please provide any further comments you may have on this variable)*

________________________________________________________________

________________________________________________________________

________________________________________________________________

________________________________________________________________

________________________________________________________________

| Page Break |  |
| --- | --- |

Q14 **Variable 4: Who are the target users for a pre-eclampsia risk screening tool?** Risk screening methods should be used by trained healthcare workers delivering routine antenatal care to pregnant women. However, the cadre responsible for providing this care may differ depending on the setting and country. Therefore, using the tool should not require the need for specialist training.

Q15 **Minimum Target:** Tool can be used by a healthcare worker delivering antenatal care, including midwives, nurses, doctors or specialist obstetricians. For some tools, laboratory staff may be required (for example if blood sample analysis is required).

- Strongly agree (5)
- Mostly agree (4)
- Neither agree nor disagree (3)
- Mostly disagree (2)
- Strongly disagree (1)
- No answer (6)

Q16 **Optimistic Target:** Same as minimum. *(Tool can be used by a healthcare worker delivering antenatal care, including midwives, nurses, doctors or specialist obstetricians. For some tools, laboratory staff may be required (for example if blood sample analysis is required)).* Plus: Tool can be used by community health workers and can be used at peripheral levels of health care in limited-resource settings.

- Strongly agree (5)
- Mostly agree (4)
- Neither agree nor disagree (3)
- Mostly disagree (2)
- Strongly disagree (1)
- No answer (6)

Q17 Additional comments *(please provide any further comments you may have on this variable)*

________________________________________________________________

________________________________________________________________

________________________________________________________________

________________________________________________________________

________________________________________________________________

| Page Break |  |
| --- | --- |

Q18 **Variable 5: What are the design and functionality requirements of a pre-eclampsia risk screening tool?** Design and functionality refer to the ease and process of setting up and operating a tool for various health care cadres across diverse settings. Consideration for internet availability is also important to maximize reach in rural and remote settings.

Q19 **Minimum Target:** Design is user friendly, simple and quick to use with minimal steps to set up or operate. Can be performed by an antenatal care worker across diverse settings.

- Strongly agree (5)
- Mostly agree (4)
- Neither agree nor disagree (3)
- Mostly disagree (2)
- Strongly disagree (1)
- No answer (6)

Q20 **Optimistic Target:** Same as minimum. *(Design is user friendly, simple and quick to use with minimal steps to set up or operate. Can be performed by an antenatal care worker across diverse settings.)*  Plus: Tool can be used offline (i.e. active internet connection not required).

- Strongly agree (5)
- Mostly agree (4)
- Neither agree nor disagree (3)
- Mostly disagree (2)
- Strongly disagree (1)
- No answer (6)

Q21 Additional comments *(please provide any further comments you may have on this variable)*

________________________________________________________________

________________________________________________________________

________________________________________________________________

________________________________________________________________

________________________________________________________________

| Page Break |  |
| --- | --- |

Q22 **Variable 6: What are the acceptability requirements of a pre-eclampsia risk screening tool?** *Acceptability refers to how a tool will be received and taken up amongst the target population and users in their respective settings.* *Availability of a tool does not guarantee uptake. The expectations and demands of local settings, users and pregnant women must be considered to facilitate strong uptake.*

Q23 **Minimum Target:** Tool can be easily and feasibly integrated into routine clinical procedures in antenatal care settings.

- Strongly agree (5)
- Mostly agree (4)
- Neither agree nor disagree (3)
- Mostly disagree (2)
- Strongly disagree (1)
- No answer (6)

Q24 **Optimistic Target:** Same as minimum. *(Tool can easily and feasibly integrate into routine clinical procedures in antenatal care settings.)*

- Strongly agree (5)
- Mostly agree (4)
- Neither agree nor disagree (3)
- Mostly disagree (2)
- Strongly disagree (1)
- No answer (6)

Q25 Additional comments *(please provide any further comments you may have on this variable)*

________________________________________________________________

________________________________________________________________

________________________________________________________________

________________________________________________________________

________________________________________________________________

| Page Break |  |
| --- | --- |

Q26 **Variable 7: What are the validation requirements for a pre-eclampsia risk screening tool?** This variable assesses whether a tool has been internally and/or externally validated. Product developers must provide proof that a tool *is based on evidence, is functional and achieves the intended use in the intended setting.*

Q27 **Minimum Target:** Tool (and its component test/s) have been developed in an evidence-based way, with robust, verifiable, peer-reviewed data demonstrating that it is valid and can accurately predict pre-eclampsia.

- Strongly agree (5)
- Mostly agree (4)
- Neither agree nor disagree (3)
- Mostly disagree (2)
- Strongly disagree (1)
- No answer (6)

Q28 **Optimistic Target:** Same as minimum. *(Tool (and its component test/s) have been developed in an evidence-based way, with robust, verifiable, peer-reviewed data demonstrating that it is valid and can accurately predict pre-eclampsia.)*  Plus: Tool has been externally validated, independent of the test developer. Tool has been externally validated in different settings and populations, including in LMICs. Tool is being updated over time to improve its performance, in response to new data or evidence.

- Strongly agree (5)
- Mostly agree (4)
- Neither agree nor disagree (3)
- Mostly disagree (2)
- Strongly disagree (1)
- No answer (6)

Q29 Additional comments *(please provide any further comments you may have on this variable)*

________________________________________________________________

________________________________________________________________

________________________________________________________________

________________________________________________________________

________________________________________________________________

| Page Break |  |
| --- | --- |

Q30 **Variable 8: What are the regulatory requirements associated with a pre-eclampsia risk screening tool?** Regulatory approval ensures that a tool is compliant with local regulations, medical standards for design and manufacture, and safety requirements.

Q31 **Minimum Target:** Tool (where applicable) has requisite regulatory approval by relevant international authorities/agencies. In countries where it is used, it is compliant with national regulatory agency standards. If tool involves a medical device, it should meet international regulatory requirements and standards, including ISO 13485:2016, and/or are in accordance with current national guidelines.

- Strongly agree (5)
- Mostly agree (4)
- Neither agree nor disagree (3)
- Mostly disagree (2)
- Strongly disagree (1)
- No answer (6)

Q32 **Optimistic Target:** Same as minimum. *(Tool (where applicable) has requisite regulatory approval by relevant international authorities/agencies. In countries where it is used, it is compliant with national regulatory agency standards. If the tool involves a medical device, it should meet international regulatory requirements and standards, including ISO 13485:2016, and/or are in accordance with current national guidelines.)*

- Strongly agree (5)
- Mostly agree (4)
- Neither agree nor disagree (3)
- Mostly disagree (2)
- Strongly disagree (1)
- No answer (6)

Q33 Additional comments *(please provide any further comments you may have on this variable)*

________________________________________________________________

________________________________________________________________

________________________________________________________________

________________________________________________________________

________________________________________________________________

| Page Break |  |
| --- | --- |

Q34 **Variable 9: What is the procurement price for a pre-eclampsia risk screening tool?** *Price is a vital consideration, and likely to vary by test type, quality and manufacturer. Prices may also vary across different countries. In addition to wholesale test costs, additional expenses such as extra equipment (e.g. transducers for imaging tests), consumables, shipping, import permits, and maintenance (if applicable) must be considered.*

Q35 **Minimum Target:** Tool is available at low or zero cost. If relevant, special tests or devices included as part of the tool are affordable to the public sector. Tool is competitively priced relative to similar technologies in low-middle income markets.

- Strongly agree (5)
- Mostly agree (4)
- Neither agree nor disagree (3)
- Mostly disagree (2)
- Strongly disagree (1)
- No answer (6)

Q36 **Optimistic Target:** Same as minimum. *(Tool is available at low or zero cost. If relevant, special tests or devices included as part of a tool are affordable to the public sector. Tool is competitively priced relative to similar technologies in low-middle income markets.)*  Plus, for any special tests included within tool: Bulk purchase discounts are available. Local manufacturing is possible.

- Strongly agree (5)
- Mostly agree (4)
- Neither agree nor disagree (3)
- Mostly disagree (2)
- Strongly disagree (1)
- No answer (6)

Q37 Additional comments *(please provide any further comments you may have on this variable)*

________________________________________________________________

________________________________________________________________

________________________________________________________________

________________________________________________________________

________________________________________________________________

| Page Break |  |
| --- | --- |

Q38 **Variable 10: What are the primary target delivery channels of a pre-eclampsia risk screening tool?** *To achieve health equity, risk screening tools should be able to be utilised at different levels of healthcare (primary, secondary and tertiary) as well as across a range of settings where women access antenatal care. Some pregnant women in rural and remote communities may only have access to antenatal care services through community outreach programs. If a tool requires the use of special tests or devices that require a laboratory, these requirements should be explicit.*

Q39 **Minimum Target:** Tool can be used in a range of health facilities that provide antenatal care services, including tertiary or secondary level hospitals, or primary antenatal care clinics.

- Strongly agree (5)
- Mostly agree (4)
- Neither agree nor disagree (3)
- Mostly disagree (2)
- Strongly disagree (1)
- No answer (6)

Q40 **Optimistic Target:** Same as minimum. *(Tool can be used in a range of health facilities that provide antenatal care services, including tertiary or secondary level hospitals, or primary antenatal care clinics.)*   Plus: Ability to offer service at community outreach settings.

- Strongly agree (5)
- Mostly agree (4)
- Neither agree nor disagree (3)
- Mostly disagree (2)
- Strongly disagree (1)
- No answer (6)

Q41 Additional comments *(please provide any further comments you may have on this variable)*

________________________________________________________________

________________________________________________________________

________________________________________________________________

________________________________________________________________

________________________________________________________________

| Page Break |  |
| --- | --- |

Q42 **Variable 11: What are the packaging requirements of a pre-eclampsia risk screening tool?** *The test/s within a tool should be easily packable to facilitate efficient shipping to any location. The packaging should reduce the risk of damage to the test during transit.  Environmental footprint and waste disposal should also be considered and limited where possible.*

Q43 **Minimum Target:** If tool includes special test/s or devices, those are easily packable. Low environmental footprint with most of the packaging recyclable.

- Strongly agree (5)
- Mostly agree (4)
- Neither agree nor disagree (3)
- Mostly disagree (2)
- Strongly disagree (1)
- No answer (6)

Q44 **Optimistic Target:** Same as minimum. *(If tool includes special test/s or devices, those are easily packable. Low environmental footprint with most of the packaging recyclable.)*   Plus: All the packaging is recyclable.

- Strongly agree (5)
- Mostly agree (4)
- Neither agree nor disagree (3)
- Mostly disagree (2)
- Strongly disagree (1)
- No answer (6)

Q45 Additional comments *(please provide any further comments you may have on this variable)*

________________________________________________________________

________________________________________________________________

________________________________________________________________

________________________________________________________________

________________________________________________________________

| Page Break |  |
| --- | --- |

Q46 **Variable 12: What are the environmental stability requirements of a pre-eclampsia risk screening tool?** *In some settings temperature control of tests or devices within a tool may not be consistently possible. They may also need to withstand a range of other climatic conditions including moisture, dust and humidity.  Therefore, they need to be reasonably durable to ensure functionality and performance.*

Q47 **Minimum Target:** If tool includes special tests or devices (including smart devices for operation), they are durable for use in all settings and can be stored and operated in a wide range of climatic conditions including heat, cold, moisture, dust and humidity.

- Strongly agree (5)
- Mostly agree (4)
- Neither agree nor disagree (3)
- Mostly disagree (2)
- Strongly disagree (1)
- No answer (6)

Q48 **Optimistic Target:** Same as minimum. *(If tool includes special tests or devices, they are durable for use in all settings and can be stored and operated in a wide range of climatic conditions including heat, cold, moisture, dust and humidity.)*

- Strongly agree (5)
- Mostly agree (4)
- Neither agree nor disagree (3)
- Mostly disagree (2)
- Strongly disagree (1)
- No answer (6)

Q49 Additional comments *(please provide any further comments you may have on this variable)*

________________________________________________________________

________________________________________________________________

________________________________________________________________

________________________________________________________________

________________________________________________________________

| Page Break |  |
| --- | --- |

Q50 **Variable 13: What are the training requirements of a pre-eclampsia risk screening tool?** *Appropriate and easy to understand training and user manuals are necessary for any specific test. Where possible, guidance for use and interpretation of results should include images and text.*

Q51 **Minimum Target:** Quick guide and user manual provided with tool, in relevant language/s used by healthcare workers in local context. Supplementary single-session, online, on-demand training (such as checklists, videos, guides) provided.

- Strongly agree (5)
- Mostly agree (4)
- Neither agree nor disagree (3)
- Mostly disagree (2)
- Strongly disagree (1)
- No answer (6)

Q52 **Optimistic Target:** Same as minimum. *(Quick guide and user manual provided with tool, in relevant language/s used by healthcare workers in local context. Supplementary single-session, online, on-demand training (such as checklists, videos, guides) provided.)*  Plus:   Quick guide and user manual provided for users, in relevant language/s for local context, plus translations into all official UN languages.

- Strongly agree (5)
- Mostly agree (4)
- Neither agree nor disagree (3)
- Mostly disagree (2)
- Strongly disagree (1)
- No answer (6)

Q53 Additional comments *(please provide any further comments you may have on this variable)*

________________________________________________________________

________________________________________________________________

________________________________________________________________

________________________________________________________________

________________________________________________________________

| Page Break |  |
| --- | --- |

Q54 **Variable 14: What are the requirements for external support during use of a pre-eclampsia risk screening tool?** *The provision of external technical support to perform troubleshooting will enhance user experience and allow for timely solutions.*

Q55 **Minimum Target:** Phone number provided to seek assistance with tool online or remotely.

- Strongly agree (5)
- Mostly agree (4)
- Neither agree nor disagree (3)
- Mostly disagree (2)
- Strongly disagree (1)
- No answer (6)

Q56 **Optimistic Target:** Built-in access to online/remote expert advice to assist tool operation via SMS, audio call or video conferencing.

- Strongly agree (5)
- Mostly agree (4)
- Neither agree nor disagree (3)
- Mostly disagree (2)
- Strongly disagree (1)
- No answer (6)

Q57 Additional comments *(please provide any further comments you may have on this variable)*

________________________________________________________________

________________________________________________________________

________________________________________________________________

________________________________________________________________

________________________________________________________________

| Page Break |  |
| --- | --- |

Q58 **Variable 15: What are the requirements of using device-based technologies within a pre-eclampsia risk screening tool?** *Device-based technologies can be used as a type of test within pre-eclampsia risk screening tools. For example, imaging tests like ultrasounds may be required to measure the pulsatility index. The minimum and optimal requirements are in relation to the test operation only and do not include additional requirements that may be necessary for sampling or sample storage.*

Q59 **Minimum Target:** If device-based technologies are used: Can be transported, assembled and performed by one person. No more than three operator steps that are not timed nor labour intensive. Reusable equipment. If non-rechargeable battery used, has back up battery power lasting minimum 45 minutes or if rechargeable integrated battery used, has minimum 5 hours on a single charge. Auto sleep/shut-off capabilities for battery saving. Results are easily readable.

- Strongly agree (5)
- Mostly agree (4)
- Neither agree nor disagree (3)
- Mostly disagree (2)
- Strongly disagree (1)
- No answer (6)

Q60 **Optimistic Target:** If device-based technologies are used: Can be transported, assembled and performed by one person. No more than one operator step that is not timed nor labour intensive. Reusable equipment using energy efficient technology. Back up battery power lasting minimum 2 hours or rechargeable integrated battery with minimum 8 hours on a single charge. Auto sleep/shut-off capabilities for battery saving. Results are easily readable.

- Strongly agree (5)
- Mostly agree (4)
- Neither agree nor disagree (3)
- Mostly disagree (2)
- Strongly disagree (1)
- No answer (6)

Q61 Additional comments *(please provide any further comments you may have on this variable)*

________________________________________________________________

________________________________________________________________

________________________________________________________________

________________________________________________________________

________________________________________________________________

| Page Break |  |
| --- | --- |

Q66 Variable 16: What are the requirements for sample types and their collection and processing procedures in a pre-eclampsia risk screening tool? *Tests included within a pre-eclampsia risk screening tool may require the need of various samples for analysis.*

Q67 **Minimum Target:** If a sample is required, collection is minimally invasive - relative to alternative methods that can be used – and requires minimal equipment to obtain sample. Minimal sample processing which does not require laboratory or cold chain.

- Strongly agree (5)
- Mostly agree (4)
- Neither agree nor disagree (3)
- Mostly disagree (2)
- Strongly disagree (1)
- No answer (6)

Q68 **Optimistic Target:** No sample required as part of tool.

- Strongly agree (5)
- Mostly agree (4)
- Neither agree nor disagree (3)
- Mostly disagree (2)
- Strongly disagree (1)
- No answer (6)

Q69 Additional comments *(please provide any further comments you may have on this variable)*

________________________________________________________________

________________________________________________________________

________________________________________________________________

________________________________________________________________

________________________________________________________________

| Page Break |  |
| --- | --- |

Q62 **Variable 17: What are the requirements of using point of care tests within a pre-eclampsia risk screening tool?** *Point of care (POC) tests can be used as a type of test within pre-eclampsia risk screening tools which provide test results at the time and place of patient care. A Rapid Antigen Test (RAT) test to detect Covid-19 is an example of a POC test. ASSURE criteria, established by the WHO, has become a benchmark for an ideal test that can be used at the point of care to ensure utilisation at all levels of the healthcare system. It stands for Affordable, Sensitive, Specific, User-friendly, Rapid, Robust, Equipment-free, and Delivered.*

Q63 **Minimum Target:** If point of care tests are used: Single use, disposable test that adheres to the ASSURE criteria.

- Strongly agree (5)
- Mostly agree (4)
- Neither agree nor disagree (3)
- Mostly disagree (2)
- Strongly disagree (1)
- No answer (6)

Q64 **Optimistic Target:** Same as minimum. *(If point of care tests are used: Single use, disposable test that adheres to the ASSURE criteria.)* Plus: Test is biodegradable or recyclable.

- Strongly agree (5)
- Mostly agree (4)
- Neither agree nor disagree (3)
- Mostly disagree (2)
- Strongly disagree (1)
- No answer (6)

Q65 Additional comments *(please provide any further comments you may have on this variable)*

________________________________________________________________

________________________________________________________________

________________________________________________________________

________________________________________________________________

________________________________________________________________

| Page Break |  |
| --- | --- |

Q70 **Variable 18: What is the clinical specificity and sensitivity of a pre-eclampsia risk screening tool?** *Having a high sensitivity for screening tests is essential to capture the greatest proportion of true positives out of all people with the condition. Specificity refers to the proportion of true negatives out of all subjects who do not have the condition. Using maternal factors only for pre-eclampsia risk screening – such as the NICE guidelines – was reported to detect 39% of women experiencing pre-eclampsia <37 weeks with a false-positive rate of 10.2%. By comparison, the FMF model at 11-13 weeks of gestation - using maternal factors, PlGF, MAP and UtA-PI - reported a sensitivity of 90% for early pre-eclampsia, at a screen positive rate (SPR) of 10%.*

Q71 **Minimum Target:** Sensitivity >70%. Specificity >60%.

- Strongly agree (5)
- Mostly agree (4)
- Neither agree nor disagree (3)
- Mostly disagree (2)
- Strongly disagree (1)
- No answer (6)

Q72 **Optimistic Target:** Sensitivity >90%. Specificity >60%.

- Strongly agree (5)
- Mostly agree (4)
- Neither agree nor disagree (3)
- Mostly disagree (2)
- Strongly disagree (1)
- No answer (6)

Q73 Additional comments *(please provide any further comments you may have on this variable)*

________________________________________________________________

________________________________________________________________

________________________________________________________________

________________________________________________________________

________________________________________________________________

| Page Break |  |
| --- | --- |

Q83 **Variable 19: What are the safety requirements of a pre-eclampsia risk screening tool?** *There should be no safety concerns associated with use of a tool.*

Q84 **Minimum Target:** Target Population: Tool will have no or minimal adverse health or safety outcomes for pregnant women. The test/s within a tool has/have no teratogenic or abortive properties posing risk to the fetus or neonate. Target Users: Standard biosafety requirements to be followed by target users. No adverse health or safety outcomes for target user.

- Strongly agree (5)
- Mostly agree (4)
- Neither agree nor disagree (3)
- Mostly disagree (2)
- Strongly disagree (1)
- No answer (6)

Q85 **Optimistic Target:** Same as minimum. (*Target Population: Tool will have no or minimal adverse health or safety outcomes for pregnant women. The test/s within a tool has/have no teratogenic or abortive properties posing risk to the fetus or neonate. Target Users: Standard biosafety requirements to be followed by target users. No adverse health or safety outcomes for target user.)*

- Strongly agree (5)
- Mostly agree (4)
- Neither agree nor disagree (3)
- Mostly disagree (2)
- Strongly disagree (1)
- No answer (6)

Q86 Additional comments *(please provide any further comments you may have on this variable)*

________________________________________________________________

________________________________________________________________

________________________________________________________________

________________________________________________________________

________________________________________________________________

| Page Break |  |
| --- | --- |

Q87 **Variable 20: How is the risk determined in a pre-eclampsia risk screening tool?** *This variable refers to how the test results from each specific test are combined in a tool. A quantifiable risk score or value will better guide clinical practice.*

Q88 **Minimum Target:** Tool stratifies women into 2 or 3 risk groups only (such as low, medium or high risk).

- Strongly agree (5)
- Mostly agree (4)
- Neither agree nor disagree (3)
- Mostly disagree (2)
- Strongly disagree (1)
- No answer (6)

Q89 **Optimistic Target:** Tool calculates a specific quantifiable risk score or value for an individual woman. Risk assessment can be updated during the course of pregnancy.

- Strongly agree (5)
- Mostly agree (4)
- Neither agree nor disagree (3)
- Mostly disagree (2)
- Strongly disagree (1)
- No answer (6)

Q94 Additional comments *(please provide any further comments you may have on this variable)*

________________________________________________________________

________________________________________________________________

________________________________________________________________

________________________________________________________________

________________________________________________________________

| Page Break |  |
| --- | --- |

Q91 **Variable 21: How will the results be displayed in a pre-eclampsia risk screening tool?** *Results must clearly show a level or score of risk of pre-eclampsia that is able to be easily understood and acted upon healthcare workers.*

Q92 **Minimum Target:** A clear high risk, low risk or invalid result with clear and simple instructions for interpretation.

- Strongly agree (5)
- Mostly agree (4)
- Neither agree nor disagree (3)
- Mostly disagree (2)
- Strongly disagree (1)
- No answer (6)

Q93 **Optimistic Target:** Same as minimum. *(A clear high risk, low risk or invalid result with clear and simple instructions for interpretation.)* Plus: Quantifiable value/risk score is provided.

- Strongly agree (5)
- Mostly agree (4)
- Neither agree nor disagree (3)
- Mostly disagree (2)
- Strongly disagree (1)
- No answer (6)

Q98 Additional comments *(please provide any further comments you may have on this variable)*

________________________________________________________________

________________________________________________________________

________________________________________________________________

________________________________________________________________

________________________________________________________________

| Page Break |  |
| --- | --- |

Q95 **Variable 22: What are the time to result requirements of a pre-eclampsia risk screening tool?** *Time to result should be as short as possible and in line with the manufacturer’s recommendations. If a tool includes multiple tests, these will each require time to obtain results, which then need to be inputted into the tool.*

Q100 **Minimum Target:** Tool produces a result within a single antenatal care visit.

- Strongly agree (5)
- Mostly agree (4)
- Neither agree nor disagree (3)
- Mostly disagree (2)
- Strongly disagree (1)
- No answer (6)

Q97 **Optimistic Target:** Tool produces a result immediately.

- Strongly agree (5)
- Mostly agree (4)
- Neither agree nor disagree (3)
- Mostly disagree (2)
- Strongly disagree (1)
- No answer (6)

Q90 Additional comments *(please provide any further comments you may have on this variable)*

________________________________________________________________

________________________________________________________________

________________________________________________________________

________________________________________________________________

________________________________________________________________

| Page Break |  |
| --- | --- |

Q99 **Variable 23: What are the tool recommendation requirements of a pre-eclampsia risk screening tool?** *A tool that not only provides a test result but also an appropriate recommendation for clinical management will aid in improving health outcomes. For example, WHO recommends that women at moderate or high-risk of developing pre-eclampsia should be offered low-dose aspirin.*

Q96 **Minimum Target:** Tool provides a risk level or score only.

- Strongly agree (5)
- Mostly agree (4)
- Neither agree nor disagree (3)
- Mostly disagree (2)
- Strongly disagree (1)
- No answer (6)

Q101 **Optimistic Target:** Same as minimum. *(Tool provides a risk level or score only.)*  Plus: Tool provides patient management recommendations, based on evidence-based guidelines, according to the risk level or score.

- Strongly agree (5)
- Mostly agree (4)
- Neither agree nor disagree (3)
- Mostly disagree (2)
- Strongly disagree (1)
- No answer (6)

Q102 Additional comments *(please provide any further comments you may have on this variable)*

________________________________________________________________

________________________________________________________________

________________________________________________________________

________________________________________________________________

________________________________________________________________

| Page Break |  |
| --- | --- |

Q103 **Variable 24: What are the data input requirements of a pre-eclampsia risk screening tool?** *Methods of data input that are more time efficient are preferable. Consideration for offline storage of data and synchronization when internet becomes available is important to accommodate settings with limited internet.*

Q104 **Minimum Target:** If tool requires data entry, manual data entry is performed by target users.

- Strongly agree (5)
- Mostly agree (4)
- Neither agree nor disagree (3)
- Mostly disagree (2)
- Strongly disagree (1)
- No answer (6)

Q105 **Optimistic Target:** Integration/ utilizing electronic patient data from existing health information systems. Data can be stored on database when offline and synchronized once internet is available.

- Strongly agree (5)
- Mostly agree (4)
- Neither agree nor disagree (3)
- Mostly disagree (2)
- Strongly disagree (1)
- No answer (6)

Q106 Additional comments *(please provide any further comments you may have on this variable)*

________________________________________________________________

________________________________________________________________

________________________________________________________________

________________________________________________________________

________________________________________________________________

| Page Break |  |
| --- | --- |

Q107 **Variable 25: What are the data security and privacy requirements of a pre-eclampsia risk screening tool?** *These requirements will ensure certain procedures are in place to preserve data integrity, identify and mitigate risks and establish relevant security processes.*

Q108 **Minimum Target:** If tool stores patient sensitive information, it operates under secure connectivity which meets data protection and regulations of individual countries to avoid loss and corruption of sensitive data, and mitigate cyberattacks, whether data are at rest or in transmission. Tool minimizes as much as possible the use of sensitive data.

- Strongly agree (5)
- Mostly agree (4)
- Neither agree nor disagree (3)
- Mostly disagree (2)
- Strongly disagree (1)
- No answer (6)

Q109 **Optimistic Target:** Same as minimum. *(If tool stores patient sensitive information, it operates under secure connectivity which meets data protection and regulations of individual countries to avoid loss and corruption of sensitive data, and mitigate cyberattacks, whether data are at rest or in transmission. Tool minimizes as much as possible the use of sensitive data.)*

- Strongly agree (5)
- Mostly agree (4)
- Neither agree nor disagree (3)
- Mostly disagree (2)
- Strongly disagree (1)
- No answer (6)

Q110 Additional comments *(please provide any further comments you may have on this variable)*

________________________________________________________________

________________________________________________________________

________________________________________________________________

________________________________________________________________

________________________________________________________________

| Page Break |  |
| --- | --- |

End of Block: Section 2: Rating your agreement with the Target Product Profiles

Start of Block: Section 3: Additional information to include in the Target Product Profiles

Q97 Are there any variables or information NOT currently in the TPP, that you think should be added?

________________________________________________________________

End of Block: Section 3: Additional information to include in the Target Product Profiles
