# Supplementary material for: Expert consensus on pre-eclampsia risk screening tools for low- and middle-income countries: Development of a new Target Product Profile
Source: PLOS Glob Public Health. 2026 Mar 2;6(3):e0005766. doi: 10.1371/journal.pgph.0005766 (PMC12952618; doi:10.1371/journal.pgph.0005766)
Supplement: S4 Appendix — (DOCX) [file pgph.0005766.s004.docx]

## S4 Appendix: Countries represented by stakeholder groups

| **Country income level** | Interviews | Survey |
| --- | --- | --- |
| *High income* | 11 (42.3) | 39 (46.4) |
| *Australia* | 3 | 12 |
| *Austria* | - | 1 |
| *Belgium* | - | 1 |
| *Finland* | 1 | - |
| *Greece* | - | 1 |
| *Israel* | - | 2 |
| *Italy* | - | 1 |
| *Japan* | - | 1 |
| *Luxembourg* | - | 1 |
| *Netherlands* | - | 1 |
| *Singapore* | - | 1 |
| *Switzerland* | 1 | 4 |
| *UK* | 3 | 7 |
| *USA* | 3 | 6 |
| *Upper middle income* | 2 (7.7) | 8 (9.5) |
| *Argentina* | 1 | 2 |
| *Guatemala* | - | 1 |
| *Mexico* | - | 2 |
| *Peru* | - | 1 |
| *South Africa* | 1 | 2 |
| *Lower middle income* | 11 (46.2) | 31 (36.9) |
| *Comoros* | - | 1 |
| *Cote d’Ivoire* | - | 1 |
| *Ethiopia* | - | 1 |
| *Ghana* | 1 | 2 |
| *India* | 2 | - |
| *Kenya* | 2 | 7 |
| *Lao* | 5 | - |
| *Myanmar* | - | 1 |
| *Nepal* | - | 1 |
| *Nicaragua* | - | 2 |
| *Nigeria* | 1 | 6 |
| *Papua New Guinea* | - | 6 |
| *Pakistan* | 1 | - |
| *Philippines* | - | 1 |
| *Senegal* | - | 1 |
| *Zambia* | - | 1 |
| *Low income* | 1(3.9) | 6 (7.1) |
| *Demographic Republic of the Congo* | - | 2 |
| *Mozambique* | 1 | 3 |
