# Supplementary material for: Expert consensus on pre-eclampsia risk screening tools for low- and middle-income countries: Development of a new Target Product Profile
Source: PLOS Glob Public Health. 2026 Mar 2;6(3):e0005766. doi: 10.1371/journal.pgph.0005766 (PMC12952618; doi:10.1371/journal.pgph.0005766)
Supplement: S7 Appendix — (DOCX) [file pgph.0005766.s007.docx]

## S7 Appendix: Target Product Profile describing tools for pre-eclampsia risk screening

**1. Variables describing the general characteristics of the risk screening tool.**

| **Variable** | **Minimum**  *The minimal target should be considered as a potential go/no go decision point.* | **Optimistic**  *The optimistic target should reflect what is needed to achieve broader, deeper, quicker global health impact.* | **Annotations**  *For all parameters, include here the* ***source data used and rationale*** *for why this feature is important.* |
| --- | --- | --- | --- |
| **General Characteristics** | | | |
| **Intended Use** | A tool for screening pregnant women to identify those at increased risk of developing pre-eclampsia.  Tool can be used throughout pregnancy, including in the first trimester. | Same as minimum.  Plus:  Tool can predict and distinguish between preterm and term pre-eclampsia. | WHO currently recommends that pregnant women at moderate to high-risk of developing pre-eclampsia should receive prophylaxis with daily low-dose (75mg) oral aspirin, ideally commencing before 20 weeks’ gestation.^1^ These recommendations vary between guidelines.^2^  Low-dose aspirin reduces the risk of pre-eclampsia (RR 0.82, 95% CI 0.77 to 0.88), fetal or neonatal death (RR 0.85, 95% CI 0.76 to 0.95) and preterm birth (RR 0.91, 95% CI 0.87 to 0.95).^3^  Preterm pre-eclampsia is associated with more severe health outcomes and may require earlier intervention to reduce the risk of developing the disease. Thus, risk screening for pre-eclampsia, as early in pregnancy as possible - ideally before 20 weeks’ gestation – permits the timely initiation of preventative care (e.g. low-dose aspirin) and more frequent clinical monitoring. Term pre-eclampsia is more common and therefore risk screening may allow for increased monitoring.    However, some women will not have access to antenatal services in early pregnancy. Therefore, the tool should predict risk of pre-eclampsia, regardless of gestational age. |
| **Target Population** | Women, adolescent girls, and transgender and gender diverse people who are pregnant and without a diagnosis of pre-eclampsia.***** | Same as minimum. | It is recommended that risk screening for pre-eclampsia would be conducted for all pregnant women.  Appropriate conduct and procedures (e.g. consent) should be adhered to when risk screening for adolescent girls who are minors. |
| **Target Countries** | Low- and Middle-Income countries, particularly those with high rates of: pre-eclampsia, high-risk populations (e.g. certain ethnic backgrounds) or poor maternal and neonatal health outcomes due to pre-eclampsia. | Same as minimum. | Pre-eclampsia can affect women in any setting or population. The highest incidences of pre-eclampsia are observed in African, European and South-East Asian regions with rates between 5.1% - 5.6%.^4^ However, the impact of pre-eclampsia on maternal mortality is greatest in sub-Saharan African and South Asia and it is the leading cause of maternal death in Latin America.^5^  As such, risk screening methods must be applicable and meet the demands (e.g. electricity, internet, staffing) of LMIC contexts. |
| **Target End Users** | Tool can be used by any skilled health personnel delivering antenatal care, including midwives, nurses, doctors or specialist obstetricians. | Same as minimum.  Plus:  Tool can be used by community health workers and can be used at peripheral levels of healthcare services.  Tool can be used by pregnant women for self-sampling, if feasible. | Risk screening methods should be used by skilled health personnel delivering routine antenatal care to pregnant women. However, the cadre responsible for providing this care may differ depending on the setting and country. Therefore, using the tool should not require highly specialized training.  Community health workers (CHWs) are often utilised to ensure that women in rural or remote communities can access key healthcare interventions.^6^ CHWs strengthen antenatal care by identifying pregnant women, diagnosing selected pregnancy-related conditions, and providing health promotion education.^7^ CHW scope of practice can vary across settings, hence local approvals must be sought if CHWs are to perform risk screening.  The terminology to describe CHWs may differ depending on country. |
| **Design and functionality** | Design is user friendly, simple and quick to use with minimal steps to set up and operate.  Tool can be used offline (i.e. active data/internet connection not required).  Risk screening can be performed at a single antenatal care visit, although some test results may require later review. | Same as minimum.  Plus:  Tool can be integrated with other routine tests conducted during antenatal care. | Across countries, a range of healthcare worker cadres deliver antenatal care services. The tool needs to be simple and user-friendly to ensure optimal use by any cadres.  Continuous internet connection is not always available in some settings, such as rural and remote locations.  Offline use must still retain optimal functionality. |
| **Primary Target Delivery Channel** | Tool can be used in a range of health facilities that provide antenatal care services, including tertiary or secondary level hospitals, or primary antenatal care clinics as well as community outreach settings where formal ANC services are offered. | Same as minimum.  Plus:  Ability to use tool within the home. | To achieve health equity, risk screening tools should be able to be used across a range of settings where women access antenatal care, as well as different levels of healthcare (primary, secondary and tertiary).  Some pregnant women in rural and remote communities may only have access to antenatal care services through community outreach programs. These programs, led by skilled health personnel, deliver antenatal care services through a mobile clinic. Given the limited infrastructure and skilled health personnel in these settings, clear and easy-to-follow referral and case-escalation pathways should be discussed and planned as part of risk screening. |
| **Acceptability and availability** | Tool deemed appropriate for use by women and target end users in target delivery channels.  Tool is easily integrated in antenatal care workflows. | Same as minimum.  Plus:  Tool deemed appropriate for use by women and target end users in home settings. | Availability of a tool does not guarantee uptake.  The tool should be acceptable by both pregnant women and target end users. Thus, the expectations and demands of local settings, policies, users and pregnant women must be considered, to ensure it is well-received and implemented into routine practices within a setting. |
| **Tool Validation** | Tool (and its components) have been developed in an evidence-based way, with robust, verifiable, peer-reviewed data demonstrating that it is valid and can accurately predict pre-eclampsia.  Tool has been externally validated in different settings and populations to those settings used during development of the tool, including in limited-resource settings. | Same as minimum. | The TPP minimum target ensures that principles of content validity and construct validity are followed.^8^ Product developers must provide proof that a tool is based on evidence, is functional, and achieves the intended use for the intended setting. They must also provide a quality certificate and registration status.  Demonstrated external validity ensures that the tool performs well within and across different contexts.  When limited-resource settings have been included in the validation process, it increases usability and integration into clinical practice/guidelines in those settings. |
| **Training Requirements** | Quick guide and user manual provided with tool, in relevant language/s for local context.    Supplementary single-session, online, on-demand training (such as checklists, videos, guides) available. | Same as minimum.  Plus:  Quick guide and user manual provided, in relevant language/s for local context, plus translations into all official UN languages.  If video tutorials are used, they are in local languages with culturally sensitive infographics and closed captions. | Each tool component will have its own specific training requirements which must be adhered to.    Appropriate and easy to understand training and user manuals are necessary for any specific test.  Where possible, guidance for use and interpretation of results should include images and text. |
| **Regulation** | Tool (where applicable) has requisite regulatory approval by relevant international authorities/agencies and/or local government bodies such as Ministry of Health.  In countries where it is used, it is compliant with national regulatory agency standards.  If the tool involves a medical device or point of care test, it should meet international regulatory requirements and standards, including ISO 13485:2016 and ISO 15189:2022 respectively, and/or are in accordance with current national guidelines. | Same as minimum. | Approval from regulatory agencies will ensure that the tool is compliant with local regulations, medical standards for design and manufacture, and safety requirements.^9^    ISO 13485:2016^10^ and ISO 15189:2022^11^ standards will ensure that devices or tests chosen will satisfy international requirements. |
| **Packaging** | All tool components and their relevant materials/consumables are easily packable.  Low environmental footprint with most of the packaging biodegradable and/or recyclable. | Same as minimum.  Plus:  All the packaging is biodegradable and/or recyclable. | Tool components and their materials and consumables should be easily packable to facilitate efficient shipping to any location. The packaging should reduce the risk of damage to the test during transit.  Environmental footprint should be considered and limited where possible, to address environmental concerns. In many settings, recycling pathways are not available, and disposal occurs into the environment in a non-controlled manner. Biodegradable materials will ensure more environmentally friendly disposal mechanisms.  Inclusion of biodegradable packaging should not impact shelf life of products. |
| **Safety** | Target Population  Tool (and its components) will have minimal adverse health effects for pregnant women.  Tool (and its components) have no teratogenic or abortive properties that might pose a risk to the fetus or neonate.    Target Users  Standard biosafety requirements to be followed by target end users.  No adverse health or safety outcomes for target end users. | Same as minimum.  Plus:  Target Population  Tool (and its components) will have no adverse health effects for pregnant women. | There have been no documented reports of adverse fetal effects for diagnostic ultrasonography procedures, including duplex Doppler imaging. However, it is advised that ultrasound imaging be performed efficiently and only when clinically indicated to minimise fetal exposure risk using the keeping acoustic output levels As Low As Reasonably Achievable (commonly known as ALARA) principle.^12^    Biomarker testing (including DNA analysis) via blood sampling or urinalysis is widely used to determine risk for various antenatal conditions and is considered safe for mother and foetus.  Some tests, such as DNA analysis via direct tissue sampling may have other risks to consider, depending on the sampling site. |
| ***Tool outputs*** | | | |
| **Results** | A clear risk category (e.g.: high, low, indeterminate) with clear and simple instructions for interpretation, is displayed.  Risk calculation is via stratification into risk groups. | Same as minimum.  Plus:  A quantifiable value with clear and simple instructions for interpretation, is displayed.  Risk calculation generates a risk score/value on a continuous scale.  Risk assessment can be updated during the course of the pregnancy. | Results must clearly show a level or risk score for developing pre-eclampsia.    Ideally, the result is easily understood and able to be acted upon by healthcare personnel.  A traffic light system corresponding to risk group is an effective way to aid in interpretation of results and should be considered.  The calculation of risk refers to how the results of each test used as part of the tool are combined to give the overall risk value or score.    A specific risk value/score (e.g. a risk of 2 in 100) is useful to clinicians and women. Some established pre-eclampsia risk screening tools use such an approach.^13^ |
| **Clinical Sensitivity and Specificity** | Sensitivity >70%    Specificity >75% | Sensitivity >80%  Specificity >90% | Current standard of care varies across settings. There is heterogeneity in recommended risk screening methods across international and national guidelines.^14-16^ Most recommended risk screening methods, including maternal history-based check lists, like those from NICE or WHO, and multi-parametric algorithms such as FMF risk screening, favour “rule-in” tests, with a higher specificity than sensitivity.^17^ This ensures fewer false positives are identified, and that detection of high risk is more trustworthy.  Having a high specificity will minimize unnecessary referrals to higher level facilities and not cause excessive burdens on the health system. |
| **Tool Recommendations** | Tool provides patient management recommendations that are linked to the risk classification or score, based on evidence-based recommendations. | Tool provides patient management recommendations that are linked to the risk classification or score, based on evidence-based international or national guidelines, (e.g. referral, follow-up, treatment, monitoring). | WHO recommends that women at high-risk of developing pre-eclampsia should be offered low-dose aspirin.^14^ WHO also recommends calcium supplementation to prevent pre-eclampsia in women with low calcium intake.^18^  Additional monitoring is required once women begin these preventative therapies and therefore the availability of required resources (staffing, medical supplies/devices) must be considered such as availability of anti-hypertensives for women with chronic or gestational hypertension. |

******* *The target population will be referred to as women throughout the TPP.*

**2. Variables describing the tool components of the risk screening tool. ****

| **Variable** | **Minimum**  *The minimal target should be considered as a potential go/no go decision point.* | **Optimistic**  *The optimistic target should reflect what is needed to achieve broader, deeper, quicker global health impact.* | **Annotations**  *For all parameters, include here the* ***source data used and rationale*** *for why this feature is important.* |
| --- | --- | --- | --- |
| **Tool Components** | | | |
| ***If the tool includes a biochemical test (e.g. biomarker – protein/RNA/DNA)*** | | | |
| **Test kit** | Easy to use test. This could be a laboratory-based test or a point-of-care test.  All materials required for test procedure, including companion devices/readers, reagents or other consumables (for example lancets, alcohol swabs) are included. | Point of care test that adheres to the ASSURE criteria.  All materials required for test procedure, including companion devices/readers, reagents or other consumables (for example lancets, alcohol swabs) included. | ASSURE (Affordable, Sensitive, Specific, User-friendly, Rapid and robust, equipment free and Deliverable to end-users) criteria, established by the WHO, has become a benchmark for an ideal test that can be used at the point of care to ensure utilisation at all levels of the healthcare system.^19^To mitigate environmental impacts, parts of the test are ideally biodegradable or recyclable.  To ensure that the test can be performed in all settings, all materials and consumables must be included in the test kit. |
| **Test kit storage and operating conditions** | *Storage conditions:*  Cool dry place between 2-30°C  Test materials may require refrigeration (2-8°C)  Shelf life up to 70% humidity.  *Operating conditions:*  15-30°C  15-70% humidity | *Storage conditions:*  Cool dry place between 2-40°C  Test materials do not require refrigeration.  Shelf life up to 90% humidity.  *Operating conditions:*  15-40°C  15-70% humidity | Test kits that require 2-8°C for storage and operation are difficult to implement for many settings. This requirement should be carefully considered to ensure equitable service delivery.  The stability and performance of tests must be evaluated at different environmental conditions. |
| **Test kit operational requirements and complexity** | Test can be performed by skilled health personnel and may require laboratory assistance.  No more than three steps to obtain sample. | Test can be performed by skilled health personnel.  No more than one step to obtain sample. | Operator steps should be as simple as possible for ease of use. |
| **Sample types, collection and processing** | If a sample (e.g. blood, urine, saliva) is required, collection is minimally to non-invasive relative to alternative methods that can be used – and requires minimal equipment to obtain sample.  Minimal, well-defined sample processing which requires basic laboratory personnel and/or equipment.  Samples may require refrigeration between 2-8°C for short periods. | Non-invasive sample required with minimal equipment to obtain sample.  Minimal, well-defined sample processing which does not require laboratory personnel or equipment.  Samples do not require refrigeration. | Biomarker testing using urine would facilitate implementation and uptake in low- and middle-income countries. If blood is needed, point of care tests requiring blood draw from fingerpick rather than needles are less painful and more time efficient.^20^  Laboratory facilities and personnel may be insufficient in low- and middle-income countries. If the tool requires a laboratory, these requirements should be explicit. In this instance, laboratory requirements would need to be factored into scale-up efforts.  If a blood test is required, it would be advantageous to do the test on whole blood rather than serum so that a centrifuge is not needed.  Clinical waste management pathways are not available in all settings and therefore the requirement of samples needs to be carefully considered in the interests of the environment. |
| **Companion equipment** | Small, portable, reusable tabletop device may be required to process samples for biochemical tests. | Small, portable, reusable handheld device, or no device required to process samples for biochemical tests. | Devices or readers may be required to process the samples for biochemical tests.  This companion equipment is separate to standard laboratory equipment. |
| **Operational requirements for companion equipment** | No more than three operator steps that are not labour intensive.  Results are easily readable in different lighting conditions.  Easy to clean with common disinfecting agents, following standard infection prevention and control guidelines. | No more than one operator step that is not labour intensive.  Results are easily readable in different lighting conditions.  Easy to clean with common disinfecting agents, following standard infection prevention and control guidelines. | Operator steps should be as simple as possible for ease of use. |
| **Power requirements for companion equipment** | AC adapter with surge protection for charging (tabletop/handheld) on mains power.  USB adaptor for handheld devices.  OR  Rechargeable battery, sufficient for 8 hours. | AC adapter with surge protection for charging (tabletop/handheld) on mains power.  USB adaptor for handheld devices.  AND  Rechargeable battery sufficient for 8 hours. | This variable is only relevant for companion tools (devices/readers) that are required to process samples of point of care tests.  Electricity requirements must be carefully considered as not all settings have consistent electrical supply.  USB adapter charging will allow for more practical modes of charging (e.g. via computers or cars). |
| **Time to result** | Result provided within a single antenatal care visit (<3 hours) or 1-2 days if requires processing in lab. | Result provided within <30 minutes. | Point-of-care testing reduces the time to obtain test results and expedites the diagnosis and initiation of treatment. This is particularly critical in settings with limited healthcare infrastructure and barriers to accessing quality and timely medical care. |
| **Test kit procurement price** | US$<10 per test | US$<1 per test | Price is a vital consideration – affordable pricing is essential to widespread use, particularly for LMICs.^21^ Price is likely to vary by test type, quality and manufacturer, as well as across different countries and markets.  The unit cost per test should include costs of required test consumables and/or reagents.  In addition to wholesale test costs, additional expenses such as shipping, import permits, and maintenance (if applicable) must be considered.  Local and/or regional manufacturing, as well as bundled pricing options, can affect unit price.  At time of writing, the following tests have pricing information available:  - PlGF testing for pre-eclampsia risk screening using standard laboratory equipment has been priced at US$50 per test (including machine costs, reagents, service charges, training and staff costs) in the United Kingdom (UK).^22^  - A point of care test for pre-eclampsia risk screening, using glycosylated fibronectin, has been priced at US$60 per test kit (reader and cartridge) in the UK.^23^ These tests are currently unaffordable in limited-resource settings.  Rapid diagnostic tests are widely used during pregnancy for syphilis, HIV and dual HIV and syphilis screening – in a study in Colombia, these cost US$1.03, US$1.23 and US$3.62 per test respectively (including test kits and supplies).^24^  In the WHO TPP on Point-of-Care Tests for Sexually Transmitted Infections, the minimum criteria for target price per combined/mono test for gonorrhoea, chlamydia, trichomoniasis and syphilis was <US$5, <US$5, max US$5 and <US$3 respectively.^25^  Further economic modelling and willingness to pay studies would be valuable to determine appropriate prices in different settings. |
| ***If the tool includes a biophysical test (e.g. blood pressure, medical imaging)*** | | | |
| **Test type** | Easy to use, standard of care test. | Point of care testing. | Test types can include but are not limited to weight measurement devices, blood pressure devices and Doppler ultrasound.  Certain devices such as ultrasound machines are not readily available in all settings; feasibility and appropriateness when selecting test types must be considered. |
| **Transducers, accessories and consumables** | All materials and consumables required to perform test included. | Same as minimum. | To ensure that the test can be performed in all settings, all transducers, accessories and consumables must be provided. |
| **Storage and operating conditions** | *Storage conditions:*  Storage temperature: -20°C to 60°C  Storage humidity: <95% relative humidity, non-condensing  *Operating conditions:*  Operating temperature: 10°C to 40°C  Operating humidity: 15 to 85% relative humidity, non-condensing | Same as minimum. | In some settings, temperature and humidity can vary widely. Tests therefore need to be reasonably durable in a range of environmental conditions, to ensure functionality and performance.  The stability and performance of the test must be validated for different environmental conditions.  Targets for operating and storage conditions have been provided for blood pressure and imaging devices based on market reference.^26,27^ The operating and storage conditions of other biophysical tests that may be included must be in line with current standards. |
| **Operational requirements and complexity** | Can be transported, assembled and performed by one skilled health personnel. Results may require review by specialist.  No more than three operator steps that are not labour intensive.  Reusable equipment.  Results are easily readable in different lighting conditions.  Easy to clean with common disinfecting agents, following standard infection prevention and control guidelines. | Handheld device that can be performed by one skilled health personnel. No assembly required.  No more than one operator step that is not labour intensive.  Reusable equipment.  Results are easily readable in different lighting conditions.  Easy to clean with common disinfecting agents, following standard infection prevention and control guidelines. | Operator steps should be as simple as possible for ease of use. |
| **Power requirements** | If non-rechargeable battery used, it has back up battery power lasting minimum 45 minutes.  If rechargeable integrated battery used, has minimum 8 hours on a single charge.  Auto sleep/shut-off capabilities for battery saving.  OR  AC adapter with surge protection for running (conventional/cart-based) or charging (portable/handheld) on mains power.  USB adaptor for handheld devices. | If non-rechargeable battery used, back up battery power lasting minimum 2 hours and other charging options available.  If rechargeable integrated battery used, has minimum 10 hours on a single charge.  Auto sleep/shut-off capabilities for battery saving.  OR  Device can be powered more than one way (e.g. AC adaptor and battery). | Electricity requirements must be carefully considered as not all settings have consistent electrical supply.  When running off battery power, notifications are necessary to alert the user of low battery to prevent the device shutting down while in use.  The availability of batteries to run devices in all settings must be considered.  Certain settings are beginning to power healthcare facilities via green energy. Therefore, if feasible, having both an AC adaptor as well as a rechargeable battery would be optimal.  USB adapter charging will allow for more practical modes of charging (e.g. via computers or cars). |
| **Time to result** | Tool produces a result within a single antenatal care visit (<3hours) or 1-2 days if requires review by physician. | Result provided within <30 minutes. | Point-of-care testing reduces the time to obtain test results and expedites the diagnosis and initiation of treatment. This is particularly critical in settings with limited healthcare infrastructure and barriers to accessing quality and timely medical care. |
| **Procurement Price** | Test available at a cost that is as low as sustainably possible, whilst maintaining quality.    Test is affordable across low-middle income markets.  Pricing should be transparently published and inclusive of all fees, including any for warranties, support and updates. | Same as minimum.    Plus, for any tests and their relevant consumables/accessories included within tool:  Bundled pricing available where applicable.  Local and/or regional manufacturing possible. | Price is a vital consideration, and likely to vary by test type, quality and manufacturer. Prices may also vary across different countries.  Affordability is essential to enable widespread use.    In addition to wholesale test costs, additional expenses such as extra equipment (e.g. transducers for imaging tests), consumables, shipping, import permits, and maintenance (if applicable) must be considered. |
| ***If the tool includes a digital platform (e.g. webpage, app)*** | | | |
| **Accessibility** | The tool is accessible through software/app/program on compatible devices. | Same as minimum. |  |
| **System configuration** | Available in all official UN languages.  Can display local time and local weights and measures. | Same as minimum.  Plus:  Option to customize to local official language and other country preferences. | Language can be a major barrier to the proper use of the tool and can lead to errors and misinterpretation. |
| **Compatible Devices** | The app/program/software is compatible with any device including:   - Smartphones - Tablets - Computers | Same as minimum. | Compatibility across a range of devices will allow the tool to be more user-friendly and have a wider range of application. |
| **External Support** | Frequently asked questions (FAQs), email address and chat BOT function (with automated answers) provided to seek assistance with tool online or remotely.  Available in all official UN languages. | Built-in access to online/remote expert advice to assist tool operation via SMS or audio call.  Platform available for calls and texts using data/internet connection or toll-free phone number provided.  Assistance available in local languages of the target end user. | The provision of external technical support to perform troubleshooting will enhance user experience and allow for timely solutions.  Given that internet and reception may not always be guaranteed where the tool is used, a FAQ sheet listing commonly encountered problems and steps to resolve them should be provided. |
| **Task Management** | One algorithm can be supported in one application against a common data set. | Multiple algorithms can be supported simultaneously in one application against a common data set. | Support of multiple algorithms simultaneously allows for multiple women to be assessed at the same time. This flexibility will allow for process efficiency if delays occur between patient consultations. |
| **System malfunction protection** | System malfunctions are made known to the target end user. | Same as minimum. | Transparency around the system malfunctions of the tool will ensure it can be operated optimally. |
| **Updates and versioning** | Processes in place to accommodate program changes and provide the appropriate and correct update to the user. | Same as minimum. | The ability for the tool to update over time in response to new data or evidence will enhance its performance. |

****** These TPP variables will only apply to those pre-eclampsia risk screening tools that include these components.

**3. Variables describing the data management requirements of the risk screening tool.**

| **Variable** | **Minimum**  *The minimal target should be considered as a potential go/no go decision point.* | **Optimistic**  *The optimistic target should reflect what is needed to achieve broader, deeper, quicker global health impact.* | **Annotations**  *For all parameters, include here the* ***source data used and rationale*** *for why this feature is important.* |
| --- | --- | --- | --- |
| **Data Management** | | | |
| **Data input** | If tool requires data entry, manual data entry is performed by users in minimum target end user variable.  Data can be stored on database when offline and synchronized once internet is available. | If tool requires data entry, manual data entry can be performed by CHWs in community outreach or pregnant women for a self-screening test.  Automatic upload of digital data (e.g. from compatible biosensors or medical devices)  Integration of electronic patient data from existing health information systems is possible. | Time efficient methods of data input are preferable.  The ability to store data offline, which can be synchronized once internet is available ensures that data can be captured anywhere and limits the need for paper records which may increase the risk of human errors and loss of data.  Utilizing and updating patient information on local health information systems as part of the tool will allow for a health-systems strengthening approach.  Automatic upload may require external integration of other software/apps that could be built in or third party however implementation costs should be carefully considered. |
| **Data security and privacy** | If tool stores patient sensitive information, it operates under secure connectivity which meets data protection and regulations of individual countries to avoid loss and corruption of sensitive data, and mitigate cyberattacks, whether data are at rest or in transmission. ISO 27001:2022 should be adhered to if no national data security policies exist.  Publicly available applications programming interfaces for data access protected by authentication and authorisation. At a minimum, technical standards are adhered to.  Tool minimizes as much as possible the use of sensitive data. | Same as minimum. | ISO 27001:2022^28^ standards will ensure that data integrity is preserved, risks are identified and mitigated, and relevant security processes are established.  Tiered access as described in user access will further enable data security and privacy.  International transmission of data should be minimized. |
| **User access** | Appropriate data access is provided based on specific roles. | Same as minimum. | Restricting access to specific roles (e.g. data manager, nurse) allows for enhanced data protection. |

**References:**

1. WHO recommendations on antiplatelet agents for the prevention of pre-eclampsia. Geneva: World Health Organization, 2021.

2. Rolnik Daniel L, Wright D, Poon Liona C, et al. Aspirin versus Placebo in Pregnancies at High Risk for Preterm Preeclampsia. *New England Journal of Medicine* 2017; **377**(7): 613-22.

3. Duley L, Meher S, Hunter KE, Seidler AL, Askie LM. Antiplatelet agents for preventing pre‐eclampsia and its complications. *Cochrane Database of Systematic Reviews* 2019; (10).

4. Abalos E, Cuesta C, Grosso AL, Chou D, Say L. Global and regional estimates of preeclampsia and eclampsia: a systematic review. *European Journal of Obstetrics & Gynecology and Reproductive Biology* 2013; **170**(1): 1-7.

5. Say L, Chou D, Gemmill A, et al. Global causes of maternal death: a WHO systematic analysis. *The Lancet Global Health* 2014; **2**(6): e323-e33.

6. What do we know about community health workers? A systematic review of existing reviews. Geneva: World Health Organization, 2020.

7. Abimbola O, Barbara M, Sarah B-Z, Nynke van den B. The roles of community health workers who provide maternal and newborn health services: case studies from Africa and Asia. *BMJ Global Health* 2019; **4**(4): e001388.

8. Zamanzadeh V, Ghahramanian A, Rassouli M, Abbaszadeh A, Alavi-Majd H, Nikanfar AR. Design and Implementation Content Validity Study: Development of an instrument for measuring Patient-Centered Communication. *J Caring Sci* 2015; **4**(2): 165-78.

9. WHO global model regulatory framework for medical devices including in vitro diagnostic medical devices. Geneva: World Health Organization, 2017.

10. International Organization for Standardization. ISO 13485:2016 Medical devices — Quality management systems — Requirements for regulatory purposes, 2020.

11. International Organization for Standardization. ISO 15189:2022 Medical laboratories — Requirements for quality and competence, 2022.

12. The American College of Obstetricians and Gynecologists. Guidelines for Diagnostic Imaging During Pregnancy and Lactation, 2017.

13. The Fetal Medicine Foundation. Risk assessment - Risk for preeclampsia. 2024. <https://fetalmedicine.org/research/assess/preeclampsia/first-trimester> (accessed 30 January 2024).

14. WHO recommendations on antiplatelet agents for prevention of pre-eclampsia. Geneva: World Health Organization; Licence: CC BY-NC-SA 3.0 IGO., 2021.

15. NICE guideline: Hypertension in pregnancy: diagnosis and management (NG133). London, UK; National Institute for Health and Care Excellence, 2023.

16. Poon LC, Shennan A, Hyett JA, et al. The International Federation of Gynecology and Obstetrics (FIGO) initiative on pre-eclampsia: A pragmatic guide for first-trimester screening and prevention. *Int J Gynaecol Obstet* 2019; **145 Suppl 1**(Suppl 1): 1-33.

17. Poon LC, Rolnik DL, Tan MY, et al. ASPRE trial: incidence of preterm pre-eclampsia in patients fulfilling ACOG and NICE criteria according to risk by FMF algorithm. *Ultrasound Obstet Gynecol* 2018; **51**(6): 738-42.

18. WHO recommendation on calcium supplementation before pregnancy for the prevention of pre-eclampsia and its complications. . Geneva: World Health Organization; 2020. Licence: CC BY-NC-SA 3.0 IGO., 2020.

19. Land KJ, Boeras DI, Chen X-S, Ramsay AR, Peeling RW. REASSURED diagnostics to inform disease control strategies, strengthen health systems and improve patient outcomes. *Nature Microbiology* 2019; **4**(1): 46-54.

20. Revvity. PlGF Testing Research. 2024. <https://www.revvity.com/au-en/category/pigf-testing-research> (accessed 02 September 2024).

21. Zakiyah N, Postma MJ, Baker PN, van Asselt AD. Pre-eclampsia Diagnosis and Treatment Options: A Review of Published Economic Assessments. *Pharmacoeconomics* 2015; **33**(10): 1069-82.

22. National Institute for Health and Care Excellence. PLGF-based testing to help diagnose suspected preterm pre-eclampsia. 2022. <https://www.nice.org.uk/guidance/dg49/chapter/2-The-diagnostic-tests> (accessed 23 June 2024).

23. National Institute for Health and Care Excellence. Lumella point-of-care test for assessing pre-eclampsia risk, 2022.

24. Obure CD, Gaitan-Duarte H, Losada Saenz R, et al. A comparative analysis of costs of single and dual rapid HIV and syphilis diagnostics: results from a randomised controlled trial in Colombia. *Sexually Transmitted Infections* 2017; **93**(7): 482.

25. World Health Organisation. Point-of-Care Tests for Sexually Transmitted Infections. Target Product Profiles and Research Questions., 2021.

26. Omron Wrist Blood Pressure Monitor, 2014.

27. Voluson E-Series BT15 / BT16 / BT17 / BT18 Service Manual, 2017.

28. International Organization for Standardization. ISO 27001:2022 Information security, cybersecurity and privacy protection — Information security management systems — Requirements, 2022.
