# Supplementary material for: Expert consensus on pre-eclampsia risk screening tools for low- and middle-income countries: Development of a new Target Product Profile
Source: PLOS Glob Public Health. 2026 Mar 2;6(3):e0005766. doi: 10.1371/journal.pgph.0005766 (PMC12952618; doi:10.1371/journal.pgph.0005766)
Supplement: Attachment — Submitted filename: PGPH-D-25-03890 RtR.docx [file pgph.0005766.s009.docx]

To the Editors,

Thank you for considering our paper for publication in PLOS Global Public Health and thank all reviewers for their comments. We have amended the manuscript following feedback from reviewers and have addressed all comments in detail below. We look forward to hearing your response.

Regards,

Dr Annie McDougall on behalf of all authors.

| **Journal comments** | **Response to reviewer** |
| --- | --- |
| In the ethics statement in the Methods, you have specified that verbal consent was obtained. Please provide additional details regarding how this consent was documented and witnessed, and state whether this was approved by the IRB. | We have amended the ethics statement, to provide further detail on how both written and verbal consent was collected for the interview participants.  *“A formal statement of consent was given by all participants, both written and verbally (interview participants) or electronically (survey participants), as approved by the Alfred Ethics Committee for Human Research. Interviewees first provided written consent to participate in an interview via response to the email invitation. Their consent was confirmed verbally during the interview and recorded in the interview notes.”*  Page 4-5, line 119 - 122 |
| Please amend your detailed Financial Disclosure statement. This is published with the article. It must therefore be completed in full sentences and contain the exact wording you wish to be published.  i. Please clarify all sources of financial support for your study. List the grants, grant numbers, and organizations that funded your study, including funding received from your institution. Please note that suppliers of material support, including research materials, should be recognized in the Acknowledgements section rather than in the Financial Disclosure.  ii. State the initials, alongside each funding source, of each author to receive each grant. For example: "This work was supported by the National Institutes of Health (####### to AM; ###### to CJ) and the National Science Foundation (###### to AM)." iii. State what role the funders took in the study. If the funders had no role in your study, please state: “The funders had no role in study design, data collection and analysis, decision to publish, or preparation of the manuscript.” iv. If any authors received a salary from any of your funders, please state which authors and which funders. | We have amended the financial disclosure statement as follows:  “The research was funded by The Gates Foundation (Grant INV-038938). JPV is supported by an Australian National Health and Medical Research Council Emerging Leadership Investigator Grant (2020/GNT1194248). ARAM is supported by the Lady Potter Emerging Leader Fellowship. The funders had no role in the study design, data collection and analysis, decision to publish, or preparation of the manuscript.”  Page 22, Lines 636-637 |
| Please provide separate figure files in .tif or .eps format. | Figures 1 has now been provided as a .tif file, rather than a .pdf. |
| We have noticed that you have uploaded Supporting Information files, but you have not included a list of legends. Please add a full list of legends for your Supporting Information files after the references list. | The list of supplemental data legends can be found at the end of the manuscript document, page 22, lines 643-650. |
| We note that your Data Availability Statement is currently as follows: All data is included within the manuscript and supplemental materials.    Please confirm at this time whether or not your submission contains all raw data required to replicate the results of your study. Authors must share the “minimal data set” for their submission. PLOS defines the minimal data set to consist of the data required to replicate all study findings reported in the article, as well as related metadata and methods ([https://journals.plos.org/plosone/s/data-availability#loc-minimal-data-set-definition](https://urldefense.com/v3/__https:/journals.plos.org/plosone/s/data-availability*loc-minimal-data-set-definition__;Iw!!G5ONXFxL7-lpCg!dS7MsU27acixANvuJfKEzxV2Cm76x0Nn7rD7VwDMFylagO-4QGVhUuyQni6465xWO8_DljPWsfI_lLmH9QuR7MbLbg$)). | Minimal data set from the stakeholder survey is now included as Supplemental Appendix 6. Some demographic data has been removed to de-identify responses. |
| **Reviewer 1** | **Response to reviewers** |
| 1. More details on the implementation challenges, acceptance, and sustainability (three sections wise), both at facility level and at community level. | We thank the reviewer for this feedback. The section “Clinical and Policy Implications” in our discussion touched on these issues, however we have now expanded them to show more clearly the barriers within facilities and in the community. While we agree a much longer dissection of these issues could be valuable, we have summarised these challenges and provided reference to more in-depth discussion, to maintain focus on the findings of our specific study.  “The current system often involves trying to retrofit innovative diagnostics, developed in HICs, to settings that were never considered in the development of the product. Implementation of new diagnostics into LMIC settings face unique challenges, including infrastructure and workforce constraints, procurement challenges due to reliance on imported tests or reagents, access issues due to high costs and geographic, gender and social barriers that limit care-seeking. Thus, women in LMICs continue to be left behind.  Page 15-16, lines 400 - 405. |
| 2. South-East Asia has a high burden, however, very minimum participants took part from this region. The author should address this issue, with some possible contributing factors. | We agree that having few participants from the South-East Asian region is a limitation and have provided context for this in the discussion.  *“Although participation in the survey from South‑East Asian representatives was limited despite the region’s high disease burden, two stakeholder interviews from India (approximately 8% of interviews) provided in‑depth input and informed the final TPP. The study also achieved a high response rate from other LMICs, including Africa, another region with substantial disease burden. As a result, the findings remain informative for understanding experiences and practices in high‑burden settings, although caution may be warranted when extrapolating results specifically to the broader South Asian context.”*  Page 16, line 423-430. |
| 3. The author should include fetal complications (IUGR, stillbirth) associated with pre-eclampsia in introduction. | We agree with the reviewer and have added this to the introduction.  “Hypertensive disorders of pregnancy are the second-leading cause of maternal deaths globally, the majority these are due to pre-eclampsia or eclampsia.(3) They also contribute significantly to fetal and neonatal mortality and morbidity, particularly fetal growth restriction, stillbirth and preterm birth.”  Page 3, lines 67 - 69 |
| 4. Please check the spelling (typo) in abstract (conclusion) | This has been corrected (line 53) |
| **Reviewer 2** | **Response to reviewers** |
| The manuscript would benefit from clarity on how LMIC stakeholder input influenced the final TPP. Which specific variables or targets were modified specifically based on feedback from LMIC participants? It would also be helpful to understand whether and how LMIC stakeholders were involved beyond interviews and surveys (e.g., in study design, interpretation, advisory roles, or review of the final TPP). | We thank the reviewer for their feedback. We have provided additional clarity on the role of LMIC stakeholders and how their inputs were prioritised.  “All feedback were collated, discussed by the research team and informed subsequent TPP revisions. When variables did not reach consensus, and/or there was disagreement among stakeholders, feedback from stakeholders in LMICs was prioritised, particularly for input related to ease of use and scale-up. Input from stakeholders with a role in tool development (researchers, product manufacturers) related to the structure of the TPP (as opposed to the content) was prioritised. The final TPP version was reviewed by the AIM project team before finalisation and publication.”  Page 8, lines 194-199 |
| Given the heterogeneity within regions, especially within LMIC contexts it may be helpful to provide more detail on subregions or contextual settings represented to better reflect this diversity. | We have now added the number of participants for both the survey and interviews from each country represented.  Supplemental Table S4. |
| Finally, the manuscript may benefit from a deeper discussion of the ethical implications of introducing pre-eclampsia risk screening tools in settings where referral pathways, timely access to preventative treatment, and health system capacity may be limited or inconsistent. | We have expanded on this issue in the discussion:  “The current system often involves trying to retrofit innovative diagnostics, developed in HICs, to settings that were never considered in the development of the product. Implementation of new diagnostics into LMIC settings face unique challenges, including infrastructure and workforce constraints, procurement challenges due to reliance on imported tests or reagents, access issues due to high costs and geographic, gender and social barriers that limit care-seeking. In addition, ethical implications must be considered when introducing new diagnostic tests in settings with limited or inconsistent referral pathways, preventive therapy, and health system capacity. Thus, women in LMICs continue to be left behind.”  Page 15-16, lines 400 - 405. |
| **Reviewer 3** | **Response to reviewers** |
| i. The manuscript details the consensus methodology and agreement threshold. Could the authors clarify how disagreements were managed when variables did not reach consensus, how minority perspectives were integrated, and whether responses from different stakeholder groups were weighted equally or some prioritised during consensus determination? | We thank the reviewer for their feedback. Most of our variables met consensus during the stakeholder engagement. In addition, where consensus was not met on the original TPP targets, most stakeholders agreed with what changes should be made to the targets. In the cases of disagreement among stakeholders, feedback from stakeholders living and working in LMICs was prioritised. An example of this can be seen in the results section, title Tool performance, where feedback was mixed between HIC and LMIC respondents. The feedback from LMIC respondents was given priority.  We have provided clarifying details to the methodology:  “All feedback were collated, discussed by the research team and informed subsequent TPP revisions. When variables did not reach consensus, and/or there was disagreement among stakeholders, feedback from stakeholders in LMICs was prioritised. The final TPP version was reviewed by the AIM project team before finalisation and publication.”  Page 8, lines 194 - 199 |
| ii. Can the authors clarify how the qualitative analysis was conducted in practice, including the number of coders involved, how coding disagreements were resolved, and whether thematic saturation was assessed? | The coding framework analysis has been included as Supplemental Appendix 5. Additional details have been added to the methods, to address these points:  “Interviews were conducted until thematic saturation was achieved, with ongoing analysis used to assess the emergence of new themes. Recruitment also ensured that all identified stakeholder groups were represented, even where saturation within a particular group was reached earlier.” Page 6, lines 152-155  AND  “This was intended to identify the significant points raised for each TPP variable, as well as potential development and implementation barriers. Analysis was performed by two researchers (TG and AM) using Microsoft Excel to collate findings. Disagreements were resolved via discussion, merging or splitting codes, and if required, discussion with a third researcher (KM and MFS). A combined directed and summative content analysis approach was utilised.” Page 7, lines 179 -181 |
| iii. Given that an exact response rate could not be calculated, can the authors discuss the potential implications of non-response bias on the consensus findings and how this may affect interpretation? | We have provided context for potential non-response bias, particularly in SE Asia, where there is a large disease burden. We have added to the discussion:  *“Although participation in the survey from South‑East Asian representatives was limited despite the region’s high disease burden, two stakeholder interviews from India (approximately 8% of interviews) provided in‑depth input and informed the final TPP. The study also achieved a high response rate from other LMICs, including Africa, another region with substantial disease burden. As a result, the findings remain informative for understanding experiences and practices in high‑burden settings, although caution may be warranted when extrapolating results specifically to the broader South Asian context.”*  Page 16, line 423-430. |
| iv. Given the heterogeneous stakeholder groups represented (Table 1), were all survey and interview responses weighted equally during consensus determination, or were certain stakeholder perspectives prioritised for specific variables? | We thank the reviewer for their feedback. We have provided additional clarity on the role of LMIC stakeholders and how their inputs were prioritised.  “All feedback were collated, discussed by the research team and informed subsequent TPP revisions. When variables did not reach consensus, and/or there was disagreement among stakeholders, feedback from stakeholders in LMICs was prioritised, particularly for input related to ease of use and scale-up. Input from stakeholders with a role in tool development (researchers, product manufacturers) related to the structure of the TPP (as opposed to the content) was prioritised. The final TPP version was reviewed by the AIM project team before finalisation and publication.”  Page 8, lines 194-199 |
| v. Did the authors assess whether perspectives from HIC-based stakeholders disproportionately influenced consensus outcomes, particularly for variables related to performance targets and implementation feasibility? | Yes, this was assessed by examining the findings of the survey responses for only those participants from LMICs. To combat any undue influence from HIC-based stakeholders, as mentioned above, in the case of disagreement among stakeholders, feedback from participants in LMICs was prioritised. This was particularly important for the clinical sensitivity and specificity variable. A description of this can be found in the results section under “Tool performance” Page 10 |
| vi. It may be helpful for the authors to consider including the full interview guide and complete survey instrument as supplementary materials to improve transparency and reproducibility. | These have now been added to the supplementary data (Supplemental Appendices 2 and 3). |
| vii. Can the authors carefully proofread the manuscript to address minor typographical errors and grammatical inconsistencies? e.g., “caare ” in abstract | This has been corrected (line 53) |
